# Adjuvant Systemic Immunotherapies for Resected Stage III Melanoma: A Single-Centre Retrospective Clinical Practice Review

**DOI:** 10.3390/ijms26020750

**Published:** 2025-01-17

**Authors:** Alicia Yioli Lefas, Cigdem Cinar, Shruti Sreekumar, Farrokh Pakzad, Panagiotis Koliou

**Affiliations:** 1 Department of Oncology, The Royal Surrey Hospital NHS Foundation Trust, Egerton Road, Guildford GU2 7XX, UK; 2 Breast and Melanoma Unit, The Royal Surrey Hospital NHS Foundation Trust, Egerton Road, Guildford GU2 7XX, UK

**Keywords:** melanoma, adjuvant, Stage III, pembrolizumab, nivolumab, recurrence-free survival (RFS), overall survival (OS)

## Abstract

Melanoma poses significant challenges due to its resistance to conventional therapies and increasing incidence rates. Stage III melanoma, characterised by regional lymph node involvement, has a high risk of recurrence despite surgical resection. Adjuvant immunotherapy, particularly using the PD-1 inhibitors pembrolizumab and nivolumab, has shown promising results in improving recurrence-free survival (RFS) and overall survival (OS) in Stage III melanoma patients. This retrospective analysis examined the effects of adjuvant pembrolizumab or nivolumab on patients with Stage III melanoma treated in a tertiary oncology centre. Of the 110 patients, 95 received pembrolizumab and 15 received nivolumab. The pembrolizumab completion rate was 62.1%, with 31.2% discontinuing due to disease progression or adverse effects. The nivolumab completion rate was lower at 40%, with 60% discontinuing due to toxicity or disease progression. Grade 3 or higher toxicities were observed in 17% of pembrolizumab and 53.3% of nivolumab patients. Disease progression occurred in 27.4% of pembrolizumab and 26.7% of nivolumab patients. Pembrolizumab showed a 12-month RFS of 78.9% and 24-month RFS of 77.6%, with an OS of 97.9% at 12 months. Nivolumab exhibited a 12-month RFS of 86.7% and 24-month RFS of 80%. RFS rates varied by disease stage and mutation status. Adjuvant pembrolizumab and nivolumab both demonstrate efficacy in improving RFS and OS in Stage III melanoma patients. Pembrolizumab has higher completion rates and fewer toxicities compared to nivolumab. Further studies are warranted to explore long-term outcomes and optimise treatment strategies.

## 1. Introduction

Melanoma is derived from the malignant transformation of melanocytes. It is the most aggressive malignant skin neoplasm and accounts for the majority of skin cancer deaths due to its nature and resistance to conventional therapeutic approaches [1]. Overall, in the UK, malignant melanoma is the fifth most common cancer, accounting for 4% of all new cancer cases, but the twentieth commonest cause of cancer death, accounting for 1% of all cancer deaths [1].

In 2020, an estimated 325,000 new melanoma cases (174,000 males, 151,000 females) and 57,000 deaths (32,000 males, 25,000 females) were reported globally [1]. The highest incidence rates occurred in Australia/New Zealand (42 and 31 per 100,000 person-years for males and females, respectively), followed by Western Europe (19 per 100,000 person-years for both genders), North America (18 and 14 per 100,000 person-years for males and females, respectively), and Northern Europe (17 and 18 per 100,000 person-years for males and females, respectively) [1]. Rates were significantly lower in Africa and Asia, often below 1 per 100,000 person-years. If these trends persist, the projected incidence of melanoma would rise to 510,000 and account for 96,000 deaths by 2040; a 50% and 68% increase, respectively [1].

Fortunately, most melanoma cases are detected in early stages, and surgical resection remains the standard treatment for localised cutaneous melanoma [2,3].

### 1.1. Molecular Aberrations Associated with Melanoma

#### 1.1.1. BRAF

Approximately 40–60% of melanomas harbour a BRAF mutation [4,5]. BRAF, a proto-oncogene encoding a serine/threonine protein kinase, is a key component of the RAS-RAF-MEK-ERK signalling pathway, which regulates cell growth and proliferation [4,5]. Activating mutations in BRAF render it a constitutively active monomer, driving unchecked cell proliferation that contributes to tumorigenesis. The V600E missense mutation, which replaces valine with glutamic acid, accounts for approximately 90% of activating BRAF mutations and constitutes approximately 50% of all metastatic melanomas [4]. The second most common mutation, V600K, results in a valine-to-lysine substitution. Research shows that in the radial growth phase, i.e., the early phase, of melanoma formation, the mutation rate in BRAF is as low as 10%, suggesting that it is not crucial for melanoma initiation [6]. Conversely, in the vertical growth phase, i.e., the phase where the cancer cells invade the dermis and can metastasise, the mutation rate is 60–70%, implicating this oncogenic mutation in cancer progression [6].

#### 1.1.2. NRAS

Mutations in the NRAS GTPase are the second most common mutation noted in melanoma after BRAF, observed in 15–20% of melanomas [7]. Activating mutations are most commonly observed in codons Q60/61 and G12/13 [7]. NRAS is another key component of the RAS-RAF-MAPK signalling pathway. Simultaneous mutations in both NRAS and BRAF are rare.

#### 1.1.3. PI3K-AKT/PTEN

The PI3K-AKT pathway is an independent signalling mechanism that contributes to cell proliferation and survival [8]. The constitutive activation of PI3K-AKT confers a competitive growth advantage, promoting proliferation and metastasis [8]. This pathway can acquire gain-of-function alterations through point mutations or changes in chromosome copy number affecting key genes, such as PIK3CA and AKT1 [8]. Analyses of melanoma tumour samples reveal only a 3% PI3K missense mutation rate, suggesting a limited direct role in melanoma development and progression [9]. However, the PI3K-AKT pathway can become hyperactivated through alternative mechanisms, including activating NRAS mutations or a loss of inhibition due to PTEN inactivation [9].

#### 1.1.4. Other Molecular Aberrations Implicated in Melanoma

The tumour suppressor gene p53 is activated by cellular stress or DNA damage to trigger apoptosis, but its role in melanoma is uncertain. While wild-type p53 expression is generally preserved, melanoma cells resist apoptosis despite cytotoxic stress, suggesting impaired p53 function [10,11,12,13]. Similarly, CDK4, a proto-oncogene facilitating G1-to-S phase transition, and its inhibitor p16INK4a (encoded by CDKN2A) are critical in familial melanoma. Mutations in p16INK4a are found in 25–60% of cases, while CDK4 mutations are rare [13].

Mutations in c-KIT, a tyrosine kinase receptor involved in melanocyte regulation, are common in acral lentiginous melanomas (23%) but rare in cutaneous melanomas (<2%), leading to KIT overexpression and tumorigenesis [14]. Additionally, MC1R variants heighten susceptibility to UV-induced melanoma through oxidative stress and mutations in genes like BRAF and NRAS. Variants such as Arg151Cys and Arg160Trp are strongly linked to increased risk [15,16].

Melanoma progression involves EMT, with E-cadherin downregulation and N-cadherin upregulation promoting migration, PI3K-AKT activation, and apoptosis resistance. VE-cadherin further supports systemic dissemination through vasculogenic mimicry [17].

### 1.2. Evidence for Current Clinical Practice in the Adjuvant Setting

Whilst Stage I and II disease are associated with exceptionally high 5-year survival rates (near 100% and 85%, respectfully), Stage III melanoma, characterised by regional lymph node involvement, displays an increased risk of recurrence following surgical resection and confers a 5-year survival of 75% [18].

Recent developments in adjuvant treatments have led to an improvement in both mortality rates and recurrence-free survival (RFS) for melanoma patients [3,19,20].

The combination of BRAF plus MEK inhibitors (dabrafenib plus trametinib) is licenced in the adjuvant setting only for patients with resected BRAF V600E or BRAF V600K mutant Stage III melanoma, based on the results of the COMBI-AD trial. This is the only targeted therapy directed towards a specific molecular aberration licenced in the adjuvant setting. In the COMBI-AD trial, adjuvant dabrafenib plus trametinib was associated with improved RFS compared to the placebo (52% vs. 36%) at 5 years. Grade 3 or 4 toxicities were reported in 41% of patients receiving dabrafenib and trametinib (vs 14% in the placebo group) [21]. In the treatment group, 26% experienced adverse events leading to discontinuation, 38% required a dose-reduction, and 66% required a dose interruption [21].

The checkpoint inhibitors pembrolizumab (Keytruda, Merck) and nivolumab (Opdivo, Bristol-Myers Squibb), administered as adjuvant therapies, have both been shown to significantly increase RFS in resected Stage III melanoma [19,20]. Both these immunotherapy agents are fully humanised IgG4 monoclonal antibodies against programmed death 1 (PD-1).

The New England Journal of Medicine published landmark phase 3 double-blind clinical trials in 2017 and 2018 evaluating pembrolizumab (EORTC 1325-MG/KEYNOTE-054) and nivolumab (CheckMate 238) as adjuvant therapies in patients with resected Stage III melanoma, compared to a placebo and the human IgG1 monoclonal antibody against cytotoxic T-lymphocyte antigen 4 (CTLA-4) Ipilimumab (Yervoy, Bristol Myers Squibb), respectively [19,20]. Both immunotherapy drugs were associated with improved overall survival (OS) [22,23]. Compared to ipilimumab, nivolumab displayed lower rates of grade 3 or 4 toxicities (14.4% vs. 45.9%) in the CheckMate 238 trial [19,20,24]. Pembrolizumab and nivolumab have not been compared head-to-head in the adjuvant setting. However, in the SWOG 1404 trial, pembrolizumab displayed lower grade 3 or 4 toxicity rates compared to ipilimumab (19.5% vs. 49.2%) [24]. Although not directly compared head-to-head, adjuvant pembrolizumab or nivolumab are associated with fewer Grade 3 or 4 toxicities compared to BRAF-targeted therapies. However, anti-PD-1 therapies more commonly cause chronic toxicities, particularly endocrinopathies or rheumatological toxicities [25].

Given the evolving landscape of immunotherapy in the adjuvant treatment of resected Stage III melanoma, we sought to retrospectively evaluate, in the real-world setting, the clinical outcomes of patients with resected Stage III melanoma treated with adjuvant immunotherapy at St Luke’s Cancer Centre, Royal Surrey County Hospital, UK. By building on the foundations of the aforementioned research, this paper will shed light on the potential of lasting benefits of immune checkpoint inhibitors as adjuvant therapy for Stage III melanoma and aims to inform clinical practice and guide treatment decisions.

It is important to recognise that clinicals often include highly selected populations based on strict eligibility criteria (e.g., age, comorbidities, performance status). Moreover, clinical trial participants are often less diverse, with the underrepresentation of minority groups and those from lower socioeconomic backgrounds, often due to accessibility barriers to specialised centres through which clinical trials are run. Even though adverse events are closely monitored and reported long-term, cumulative side effects may not emerge until treatments are used in broader populations in the real world. This study fills that gap by evaluating outcomes in a real-world clinical setting.

We hypothesise that, in keeping with the conclusions of the landmark clinical trials, the use of checkpoint inhibitors in the adjuvant setting for our cohort of patients with resected Stage III melanoma improves both recurrence-free survival and overall survival.

## 2. Results

### 2.1. Patients and Treatment

A total of 110 patients with resected Stage III melanoma received adjuvant immunotherapy (pembrolizumab or nivolumab) at St Luke’s Cancer Centre from November 2018 through to March 2023. A total of 95 patients (86.4%) received adjuvant pembrolizumab, whilst only 15 patients (13.6%) received adjuvant nivolumab. The demographics and baseline characteristics were similar in both groups (Table 1).

It is important to note that the small sample size (n = 15) of patients that received nivolumab introduces numerous biases, including selection bias, random errors, overestimation or underestimation of true effect size, and reduced statistical power. These biases undermine the validity and generalisability of the results, making it harder to draw accurate conclusions.

Of the 95 patients that received pembrolizumab, 35 (36.8%) received 400 mg six-weekly and the majority, 51 (53.7%), 200 mg three-weekly. Nine patients started on the three-weekly schedule and switched to the six-weekly schedule during adjuvant treatment.

In the pembrolizumab group, 59 patients (62.1%) completed 1 year of adjuvant treatment. Adjuvant pembrolizumab was discontinued in the case of 35 patients (31.2%), with the cessation of treatment arising from disease progression in 19 cases and the remainder owing to adverse effects. One patient discontinued treatment due to non-attendance. Grade 3 or above toxicities were reported in 17 patients receiving pembrolizumab (Table 2).

In the nivolumab group, six patients (40.0%) completed 1 year of adjuvant treatment. Adjuvant nivolumab was discontinued in the case of nine patients (60.0%), with the cessation of treatment arising from disease progression in two cases and the remainder owing to grade 3 or above toxicities reported in seven patients (Table 2).

### 2.2. Efficacy

In the pembrolizumab group (n = 95), disease progression was observed in 26 patients (27.4%). Of these, 12 patients (46.2%) experienced local recurrence and 14 patients (53.8%) relapsed with distal metastases. Of the total of 26 patients that relapsed, 19 patients (73.1%) progressed during adjuvant treatment and 7 patients (26.9%) progressed after completing or prematurely discontinuing adjuvant treatment.

Regarding the latter, five out of the seven patients completed one year of adjuvant pembrolizumab and then relapsed (one at their end-of-treatment scan, two one year post completion of adjuvant treatment, and two over two years later). The remaining two out of the seven patients stopped adjuvant treatment prematurely due to toxicity (Grade 3 skin rash after seven cycles of pembrolizumab administered six-weekly and Grade 3 nephritis after ten cycles of pembrolizumab administered three-weekly), and both progressed over 12 months from their initial diagnosis. At the time of analysis, 13 deaths had occurred in the pembrolizumab group, all of which had evidence of disease progression.

In the nivolumab group (n = 15), disease progression was observed in four patients (26.7%). Of these, two patients relapsed whilst undergoing adjuvant treatment with nivolumab (after 3 and 13 cycles, respectively). The other two patients relapsed after the premature cessation of adjuvant nivolumab. Of these, one patient stopped adjuvant treatment after developing Grade 3 transaminitis after a single cycle of nivolumab and then relapsed 4 months later, whilst the other patient stopped adjuvant treatment after 10 cycles and relapsed 3 years later. All cases of disease recurrence were due to disease relapse distally. No deaths had occurred in the nivolumab group at the time of analysis.

Among patients with disease progression, outcomes varied based on subsequent treatments. Twelve patients received next-line combination ipilimumab and nivolumab; four discontinued due to toxicity and seven transitioned to maintenance nivolumab. Despite this, five died. Six patients with BRAF-mutant melanoma received dabrafenib and trametinib; one remains in remission after 4 years, while three progressed and switched to next-line ipilimumab and nivolumab. Of these patients, one remains under surveillance and two died. Three patients experienced local recurrence, underwent surgical resection, and remain in remission. Five patients who progressed on pembrolizumab received no further treatment; three died from progression, and two remain under surveillance with stable disease.

#### 2.2.1. Intention-to-Treat Population

**Figure 1:**
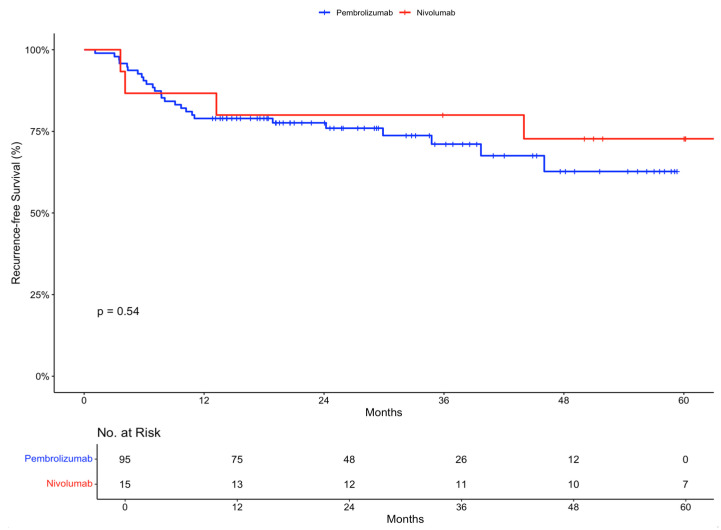
Recurrence-free survival in the intention-to-treat population stratified by treatment received.

The 12-month recurrence-free survival rate was 78.9% (95% confidence interval [CI], 71.2 to 87.6) in the pembrolizumab group and 86.7% (95% CI, 71.1 to 100) in the nivolumab group. At 24 months, the recurrence-free survival was 77.6% (95% CI, 69.6 to 86.6) and 80% (95% CI, 62.1 to 100), respectively (Figure 1).

**Figure 2:**
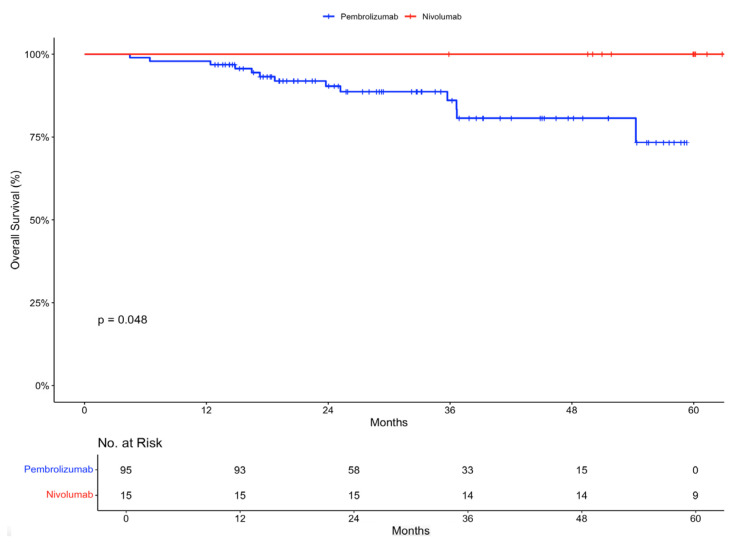
Overall survival in intention-to-treat population stratified by treatment received.

Overall survival at 12 months was 97.9% (95% CI, 95.1 to 100) in the pembrolizumab group and 100% in the nivolumab group (Figure 2).

#### 2.2.2. Recurrence-Free Survival According to Stage III Substage

**Figure 3:**
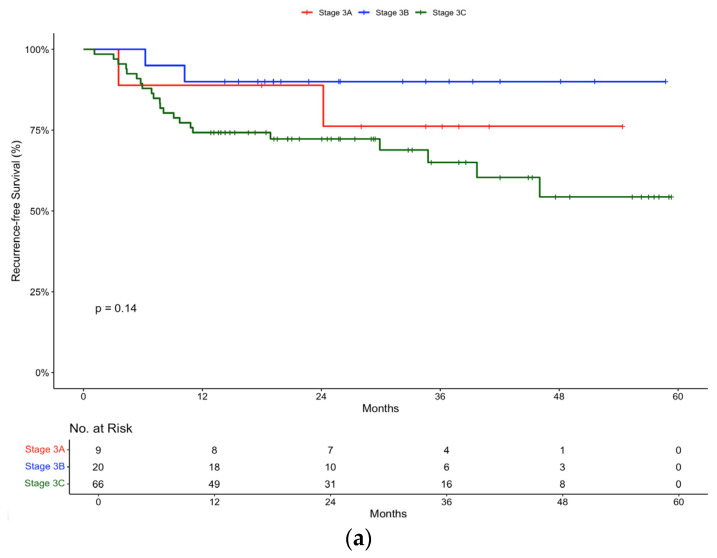

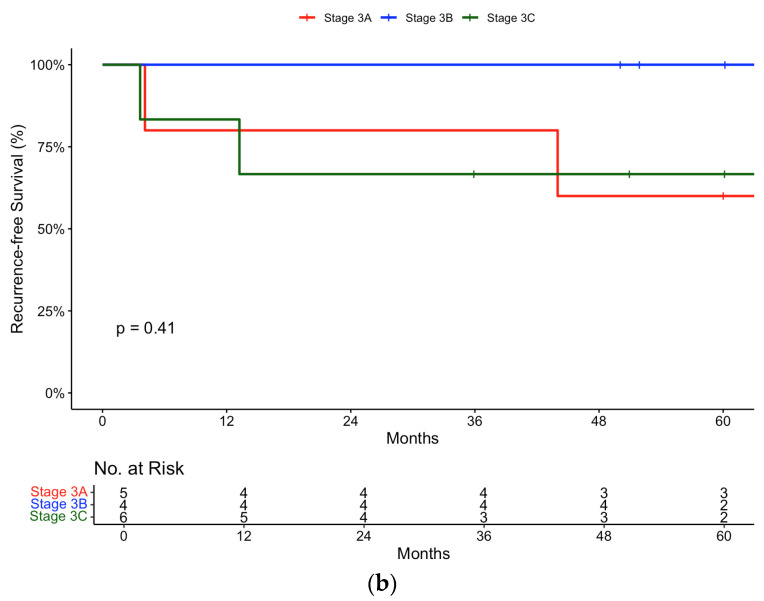
Recurrence-free survival in (**a**) pembrolizumab and (**b**) nivolumab groups stratified by Stage (IIIA, IIIB, IIIC).

In the pembrolizumab group, subgroup analysis according to disease stage revealed the following recurrence-free survival rates: a Stage IIIA recurrence-free survival of 88.9 (95% CI, 70.6 to 100) at 12 and 24 months, Stage IIIB recurrence-free survival of 90% (95% CI, 77.8 to 100) at 12 and 24 months, and Stage IIIC recurrence-free survival of 74.2% (95% CI, 64.4 to 85.6) at 12 months and 72.3% (95% CI, 62.1 to 84.1) at 24 months (Figure 3a). As expected, Stage IIIC disease conferred a lower recurrence-free survival rate. Unexpectedly, the recurrence-free survival of Stage IIIB melanoma was slightly improved compared to Stage IIIA.

In the nivolumab group, subgroup analysis according to disease stage revealed the following recurrence-free survival rates: a Stage IIIA recurrence-free survival of 80% (95% CI, 51.6 to 100) at 12 and 24 months, Stage IIIB recurrence-free survival of 100% (95% CI, 100 to 100) at 12 and 24 months, and Stage IIIC recurrence-free survival of 83.3% (95% CI, 58.3 to 100) at 12 months and 66.7% (95% CI, 37.9 to 100) at 24 months (Figure 3b). Similarly to the pembrolizumab cohort, the same trends were observed in the nivolumab cohort.

#### 2.2.3. Recurrence-Free Survival According to Mutation Status

**Figure 4:**
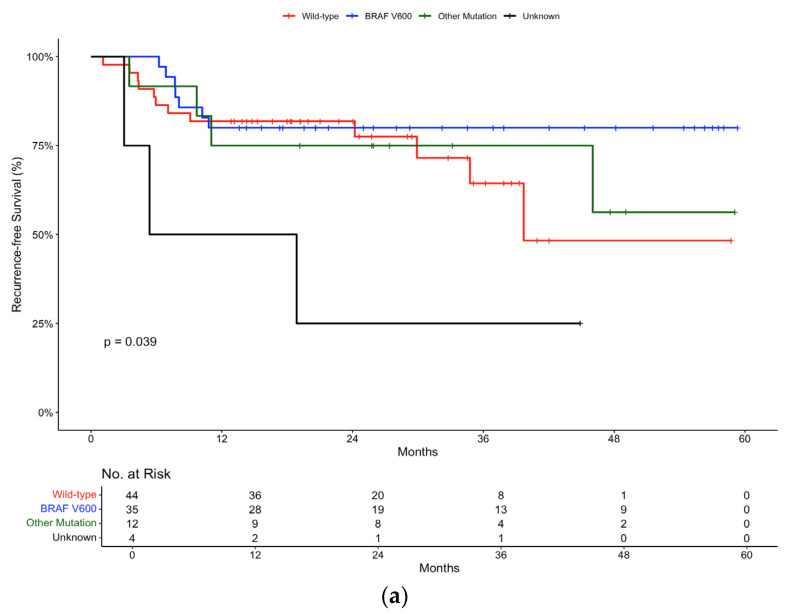

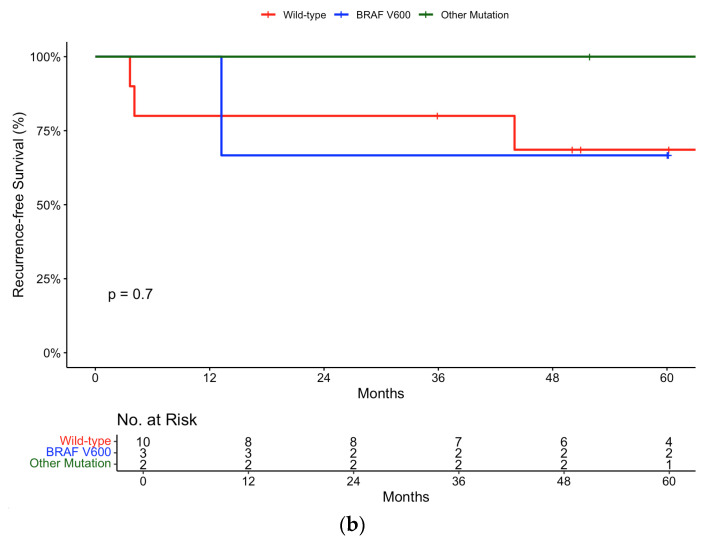
Recurrence-free survival in (**a**) pembrolizumab and (**b**) nivolumab groups stratified by mutation status.

Further subgroup analysis was carried out according to mutation status. In the pembrolizumab group, the 12-month recurrence-free survival rate was 81.8% (95% CI, 71.2 to 94) for wild-type patients, compared to 80% (95% CI, 67.8 to 94.4) for patients with a BRAF V600 mutation and 75% (95% CI, 54.1 to 100) for patients with other mutations (Figure 4a). There was minimal difference in the 12-month recurrence-free survival rates between the BRAF wild-type and BRAF-mutant populations, suggesting that pembrolizumab is equally effective, irrespective in both groups.

In the nivolumab group, the 12-month recurrence-free survival rate was 80% (95% CI, 58.7 to 100) for wild-type patients compared to 100% (95% CI, 100 to 100) for patients with a BRAF V600 (Figure 4b).

## 3. Discussion

This study provides valuable insights into the real-world application and outcomes of pembrolizumab or nivolumab administered in the adjuvant setting to patients with resected Stage III melanoma, which aligns with findings from pivotal clinical trials [19,20].

On assessment of the baseline characteristics of patients receiving either pembrolizumab or nivolumab, both groups were well-balanced, indicating a representative sample for both treatments. The majority of patients received pembrolizumab, reflecting its broader usage compared to nivolumab, which aligns with its approval and adoption in accordance with NICE guidelines [26].

In terms of efficacy, the study shows promising results. The 12-month recurrence-free survival rates were 78.9% for pembrolizumab and 86.7% for nivolumab, with comparable trends at 24 months. These results did not reach statistical significance (*p*-value 0.54) but are consistent with the outcomes reported in the landmark trials assessing both pembrolizumab and nivolumab as adjuvant therapies for Stage III melanoma [27]. It is important to note that the results from the nivolumab group need to be interpreted in the context of a small sample size (n = 15).

In KEYNOTE-054, examining adjuvant pembrolizumab vs. placebo in resected Stage III melanoma, the 12-month recurrence-free survival was quoted as 75.4% (95% CI 71.3 to 78.9) [19]. Our analysis yielded similar results without reaching statistical significance. In CheckMate 238, examining adjuvant nivolumab vs. ipilimumab in resected Stage III or IV melanoma, the 12-month recurrence-free survival was quoted as 70.5% (95% CI 66.1 to 74.5) [20]. It is important to note that CheckMate 238 included patients with Stage IV melanoma (82 out of the recruited 453 that received nivolumab). Stratifying by disease stage resulted in a 12-month recurrence-free survival rate of 72.3% (95% CI 67.4 to 76.7) for those with Stage III disease [20]. In our analysis, the 12-month recurrence-free survival rate was significantly higher.

Overall survival rates at 12 months were high, reaching 97.9% for pembrolizumab and 100% for nivolumab.

Subgroup analyses based on disease stage and mutation status also provide additional insights into treatment outcomes. Both pembrolizumab and nivolumab demonstrated efficacy across different disease stages, with varying recurrence-free survival rates. As expected, Stage IIIC disease, representing a high-risk subgroup with more extensive nodal involvement and/or ulceration, was associated with poorer outcomes. In the pembrolizumab subgroup, the recurrence-free survival rate was very similar in patients with resected Stage IIIA (88.9%) and Stage IIIB (90%).

The clinical benefit of offering adjuvant treatment in patients with Stage IIIA disease has been a controversial topic. Moncrief et al. postulated that micro-metastatic tumour burden can sub-stratify Stage IIIA [28]. In their large multi-centre study, they found that a 0.3 mm cutoff effectively distinguished IIIA melanoma patients into two subcategories with differing survival outcomes. Patients with deposits < 0.3 mm had survival rates similar to Stage IB (N0), while those with deposits ≥ 0.3 mm had significantly worse recurrence-free survival; 92% vs. 72%, respectively, at 5 years [28]. Effective risk stratification is crucial for guiding adjuvant therapy decisions, especially when balancing potential benefits against the risks of toxicity.

Additionally, in the pembrolizumab group, outcomes according to mutation status showed comparable efficacy between wild-type and BRAF V600 mutation groups, suggesting the effectiveness of checkpoint inhibitors regardless of mutation status. These results were statistically significant (*p*-value 0.039). This is in line with clinical trial data as exemplified by the pooled analysis of three clinical trials by Puzanv et al., which concluded in favour of the use of pembrolizumab in the setting of advanced melanoma, regardless of patients’ BRAF V600E/K mutation status [29].

There have been no randomised clinical trials directly comparing BRAF-targeted therapy and anti-PD1 therapy in the adjuvant setting. Other than BRAF mutation status, there are no biomarkers to select one therapy over the other. Studies indicate that while immunotherapy carries a rare risk of serious immune-related adverse effects, which can occasionally lead to chronic symptoms or mortality, the majority of toxicities are readily reversible [30]. Compared to BRAF-targeted therapy, immunotherapy demonstrates a more favourable safety profile with fewer dose interruptions and patients more likely to complete a year of adjuvant treatment [30].

The study also highlights the safety profile of these treatments. Grade 3 or higher toxicities were reported in both pembrolizumab and nivolumab groups.

In CheckMate 238, Grade 3 or above toxicities were reported in 14.4% of those in the nivolumab group. The commonest toxicities observed were those of colitis, hepatitis (rise in ALT level), and rash. In our study, a higher rate of toxicity was observed with nivolumab. The commonest observed toxicity was that of Grade 3 arthralgia. This is supported by clinical trial data where MSK symptoms were reported by approximately 40% of patients and were more prominent in PD-1 inhibitors [31]. Two patients also experienced Grade 3 pneumonitis, a potentially serious toxicity. Similarly to CheckMate 238, treatment-related deaths were observed in our study.

The higher incidence of Grade 3 or the above toxicities in our study, compared to CheckMate 238, may reflect differences in patient populations, often with more comorbidities and prior treatments which may predispose them to immune-related toxicities. Moreover, it is important to note that there may be an element of overestimation of true effect size due to the small sample size of the nivolumab group.

Regarding the toxicity profile of pembrolizumab, in our study, this was more equally distributed across organ systems. The incidence of Grade 3 and the above toxicities was lower compared to the nivolumab group but slightly higher (20% vs. 14.7%) than that quoted in the KEYNOTE-054 trial. Grade 3 arthralgia was again commonly observed, as was rash, colitis, pneumonitis, and hypophysitis. Although arthralgia was not observed as frequently in KEYNOTE-054, immune-related Grade 3 and the above adverse events included the following: endocrinopathies, colitis, hepatitis, and pneumonitis. In KEYNOTE-054, there was one treatment-related death secondary to myositis. Grade 3 myositis was also observed in our study (2.1%), but there were no treatment-related deaths.

In other real-world retrospective studies comparing adjuvant nivolumab and pembrolizumab in Stage III melanoma patients, there has been no significant difference observed in the overall occurrence of immune-related adverse effects. Gawaz et al. found that compared to younger patients, those ≥ 75 years old experienced a significantly higher proportion of skin toxicity, nephrological toxicity, and colitis [32].

The limitations of this study must be acknowledged when interpreting the findings. For instance, certain observations did not reach statistical significance in our analyses. This may be attributed to the small sample size, particularly in the nivolumab group, and thus the generalisability of these results to the broader population needs to be considered. Furthermore, as a single-centre study, the applicability of the results may further be limited.

Being a retrospective study, it is important to recognise that the data were collected at a specific point in time, which introduces certain limitations such as incomplete data, the potential evolution of data over time, and a loss of follow-up, thereby impacting the accuracy of our findings.

However, this study also had several strengths. Utilising a heterogeneous population with similar baseline characteristics in both treatment groups enhances the validity of our findings to the broader population. Additionally, conducting our research at a large tertiary centre and performing various subgroup analyses further reinforces the relevance of these results and provides deeper insights into factors influencing survival in patients receiving immunotherapy. The longitudinal follow-up of patients over a considerable period allowed for the assessment of recurrence-free survival at 12 and 24 months post-treatment initiation, providing constructive insights into the long-term efficacy of adjuvant immunotherapy.

Overall, this study reinforces the role of adjuvant pembrolizumab or nivolumab in improving outcomes for patients with resected Stage III melanoma. Despite its limitations, the findings support the integration of these immunotherapy agents into clinical practice guidelines and add to the growing evidence supporting the efficacy and safety of pembrolizumab and nivolumab as adjuvant therapies in Stage III melanoma. It has also provided an insight into the importance of personalised strategies, as efficacy is affected by disease and patient characteristics.

However, further research, particularly prospective multi-centre studies, is essential to inform treatment strategies for melanoma patients. Involving multiple centres would ensure diverse demographic groups, treatment settings, and clinical practices, better reflecting real-world conditions. Long-term prospective studies could provide valuable insights into survival rates and how immunotherapy-related toxicities affect quality of life and the potential for chronic health issues post-treatment. Lastly, incorporating biomarkers and other predictive tools is a critical unmet research need that is required to enhance patient care.

## 4. Materials and Methods

This project was registered and approved by the Associate Director of Operations at the Royal Surrey County Hospital. The audit team identified patients who were diagnosed with Stage III melanoma and received adjuvant immunotherapy. The total number of patients included in this study was 110. Of these, 95 patients received pembrolizumab, and 15 received nivolumab.

Data for this study were gathered from the following sources: Somerset Cancer Register (SCR) Oncology Clinic Letters, EPR/ARIA ePrescribing, and Electronic Patient Records—‘Surrey Safe Care’. The data collected included patient demographics (gender, age) and melanoma-specific details, including the following: the primary site of melanoma, TNM staging, Breslow’s thickness, mitotic activity, BRAF status, and LDH levels. Additionally, data were collected regarding the treatment each patient received (i.e., pembrolizumab or nivolumab) and the interval between treatment cycles (i.e., three-weekly or six-weekly for pembrolizumab and four-weekly for nivolumab). Data regarding the treatment start and end dates were collected. Regarding the latter, we documented whether the course of planned adjuvant treatment was completed in full or discontinued prematurely, either due to toxicity or disease progression. We recorded whether this occurred locally or distally for the patients that relapsed. Mortality data were collected and official death certificates corroborated the cause of death. Data and survival analysis was performed in RStudio (Version 2023.12.1+402).

## 5. Conclusions

This single-centre retrospective study reinforces the positive role of adjuvant pembrolizumab and nivolumab in improving recurrence-free survival and overall survival in patients with resected Stage III melanoma. Both treatments demonstrated efficacy across various subgroups. Toxicity profiles were generally in line with prior clinical trials, though this study observed a higher incidence of grade 3 or above toxicities with nivolumab. This study supports the real-world applicability of checkpoint inhibitors and contributes to the growing body of evidence on the use of pembrolizumab and nivolumab in the adjuvant treatment of melanoma.

## Figures and Tables

**Table 1:** Demographic and clinical characteristics of patients at baseline.

Characteristic	Pembrolizumab (n = 95)	Nivolumab (n = 15)
**Gender—no. (%)**		
Male	37 (38.9)	4 (26.7)
Female	58 (61.1)	11 (73.3)
**Age at Diagnosis—no. (%)**		
<50	17 (17.9)	2 (13.3)
50–65	37 (38.9)	9 (60.0)
≥65	41 (43.2)	4 (26.7)
**Primary Site—no. (%)**		
Head and Neck	18 (18.9)	2 (13.3)
Trunk	16 (16.8)	4 (26.7)
Back	12 (12.6)	0 (0.0)
Upper Limb	20 (21.1)	2 (13.3)
Lower Limb	26 (27.4)	7 (46.7)
Groin	3 (3.2)	0 (0.0)
**Stage—no. (%)**		
Stage IIIA	9 (9.5)	5 (33.3)
Stage IIIB	20 (21.1)	4 (26.7)
Stage IIIC	63 (66.3)	6 (40.0)
Stage IIIC with ≥4 lymph nodes	3 (3.2)	0 (0.0)
**BRAF Status—no. (%)**		
Wild type	44 (46.3)	10 (66.7)
V600E mutation	35 (36.8)	3 (20.0)
Other mutation	12 (12.6)	2 (13.3)
Unknown	4 (4.2)	0 (0.0)

**Table 2:** Grade 3 or higher toxicities reported in the pembrolizumab vs. nivolumab groups.

Event—No. (%)	Pembrolizumab (n = 19)	Nivolumab (n = 8)
Adrenal Insufficiency/Hypophysitis	2 (2.1)	0 (0.0)
Arthritis	4 (4.2)	3 (18.8)
Rash	5 (5.3)	1 (6.3)
Myositis	2 (2.1)	0 (0.0)
Myocarditis	1 (1.1)	0 (0.0)
Pneumonitis	2 (2.1)	2 (12.5)
Colitis	2 (2.1)	1 (6.3)
Nephritis	1 (1.1)	0 (0.0)
Hepatitis	0 (0.0)	1 (6.3)

## Data Availability

The original contributions presented in this study are included in the article. Further inquiries can be directed to the corresponding author.
